# Effects of Plant Functional Group Loss on Soil Microbial Community and Litter Decomposition in a Steppe Vegetation

**DOI:** 10.3389/fpls.2017.02040

**Published:** 2017-11-28

**Authors:** Chunwang Xiao, Yong Zhou, Jiaqi Su, Fan Yang

**Affiliations:** ^1^College of Life and Environmental Sciences, Minzu University of China, Beijing, China; ^2^State Key Laboratory of Vegetation and Environmental Change, Institute of Botany, Chinese Academy of Sciences, Beijing, China; ^3^Department of Ecosystem Science and Management, Texas A&M University, College Station, TX, United States; ^4^University of Chinese Academy of Sciences, Beijing, China

**Keywords:** functional group loss, soil microbial community, phospholipid fatty acid analysis, litter decomposition, steppe vegetation

## Abstract

Globally, many terrestrial ecosystems are experiencing a rapid loss of biodiversity. Continued improvements in our understanding of interrelationships between plant diversity and soil microbes are critical to address the concern over the consequences of the decline in biodiversity on ecosystem functioning and services. By removing forbs, or grasses, or, to an extreme scenario, both forbs and grasses in a steppe vegetation in Inner Mongolia, we studied how plant functional group (PFG) loss affects soil microbial community composition using phospholipid fatty acid analysis (PLFA) and litter decomposition using a litter-bag method. PFG loss significantly decreased above- and below-ground plant biomass, soil microbial biomass carbon (SMBC) and nitrogen (SMBN), but had no effect on the ratio of SMBC to SMBN. Although the ratio of fungal to bacterial PLFAs remained unaffected, PFG loss significantly reduced the amount of bacterial, fungal, and total PLFAs. PFG loss decreased litter monthly mass loss and decay constant, and such decrease was significant when both forbs and grasses were removed. Our results provide robust evidence that PFG loss in grassland ecosystem can lead to a rapid response of soil microbial activity which may affect litter decomposition and soil nutrient cycling, suggesting that the assessment of plant–microbe interactions in soils is an integral component of ecosystem response to biodiversity loss.

## Introduction

Globally, species composition and numbers in terrestrial ecosystems are being modified by anthropogenic activities, generating concerns that ecosystem functioning and services would be affected by this unprecedented loss of biodiversity (Tilman, 2000; Jenkins, 2003; Reich et al., 2012; Tilman et al., 2014). The prevailing view from the abundance literature is that loss of biodiversity decreases net primary productivity and alters plant–plant interactions, reducing aboveground ecosystem functioning (Naeem et al., 1994; Hooper and Vitousek, 1997; Cardinale et al., 2012; Tilman et al., 2014). Emerging studies have also shown that loss of biodiversity has dramatic impacts on soil microbial communities (Loranger-Merciris et al., 2006; Bardgett and Wardle, 2010; Butenschoen et al., 2011; Szanser et al., 2011; Chen et al., 2016), affecting litter decomposition rates and soil nutrient cycling and representing a mechanistic link between plant diversity and functioning of belowground systems (Zak et al., 2003; Eisenhauer et al., 2010; Bardgett and van der Putten, 2014).

Soil microorganisms are mostly heterotrophic, they acquire carbon (C) as energy from organic resources, such as leaf and root litter, root exudates, that are derived primarily from plants. As different plant species have different productivity and biochemical composition, a loss in plant diversity or plant functional groups (PFGs) is likely to alter the quantity and/or quality of these organic resources, thereby modifying the abundance, activity and diversity of soil microbial communities (Zak et al., 2003; Bardgett and Wardle, 2010; Eisenhauer et al., 2010; Lange et al., 2015). However, results from previous studies have a high degree of uncertainty as to whether species/PFG loss has a negative, neutral or positive effect on soil microbial communities. For example, some experimental studies have indicated that plant species loss leads to decreased soil microbial biomass (Eisenhauer et al., 2010), abundance and functional diversity of bacterial or fungal (Zak et al., 2003; Lamb et al., 2011; Chen et al., 2016), whereas many others have reported that responses of soil microbial communities to plant diversity/richness are insensitive (Orwin and Wardle, 2005; Loranger-Merciris et al., 2006; Berg and Smalla, 2009). Reasons for these discrepancies remain unknown, but may be ascribed to the type of ecosystems, the extent of PFG/species loss, and/or the duration of the experimental manipulation (Eisenhauer et al., 2010). In addition to the potential alteration of organic resources, species/PFG loss also modifies soil environmental conditions that indirectly affect the behavior of soil microorganisms (Lorentzen et al., 2008), thus representing additional challenges to unveil the interrelationships between plant diversity and soil microbial communities.

Generally, litter decomposition is controlled by litter quality, the composition and structure of litter decomposer community, and the physicochemical environment (Berg et al., 1993; Aerts, 1997; Salinas et al., 2011; Zhou et al., 2015). Under given environmental conditions, both litter quality and decomposers (as noted earlier) are directly linked to plant diversity (Hättenschwiler et al., 2005). A series of litter traits, such as leaf mass per area, litter nitrogen (N) and lignin concentrations, litter C/N and lignin/N ratios, have been identified as predominant controls on litter decomposability and used for predicting and modeling litter decomposition rates (Cornwell et al., 2008; Allison, 2012; Makkonen et al., 2012). As plant species differ widely in their leaf traits (Wright et al., 2004), species/PFG loss is expected to alter the quantity and quality of litter materials entering the soils, thereby affecting litter utilization by decomposers and corresponding decomposition rates (Hector et al., 2000; Hättenschwiler et al., 2005; Szanser et al., 2011). Previous studies regarding the effect of plant diversity on litter decomposition processes are mostly species-based (i.e., compare mixed-species litter to the respective single species litter) and results are inconsistent, ranging from synergistic interactions among litter species to antagonistic interactions (Gartner and Cardon, 2004; Hättenschwiler et al., 2005; Lecerf et al., 2011; Szanser et al., 2011). However, less is known about the effect of PFG loss on the decomposition rate of leaf litter from each PFG.

Grasslands, which are globally important ecosystems sustaining the livelihoods of a large proportion of the world’s human population, have been widely and experimentally used to test the effect of species/PFG loss on ecosystem functioning and services (the Cedar Creek Experiment, Tilman and Downing, 1994; the Jena Experiment, Weigelt et al., 2010). In grassland vegetation, grasses and forbs are two dominant but divergent functional groups which have different plant productivity and palatability that affect ecosystem processes differently (Díaz and Cabido, 2001). The Inner Mongolian steppe, part of the Eurasian steppe constituting the largest contiguous grassland in the world, is experiencing unprecedented degradation due to increasing numbers of livestock (Kang et al., 2007). As large herbivores feed preferentially on different plant foliage, selective overgrazing in the Inner Mongolian steppe is changing the relative abundance of grasses vs. forbs and resulting in species/PFG loss and, to an extreme scenario, bare ground (Schönbach et al., 2011). A few earlier reports have demonstrated that the loss of PFG in the Inner Mongolian steppe has dramatic impacts on soil biota and nutrients (Yuan et al., 2015; Chen et al., 2016). Continued improvement in our understanding of changes in soil microbial communities and litter decomposition rates resulting from PFG loss would enhance our ability to predict responses of belowground processes to biodiversity loss in grassland ecosystems.

The overall objective of this study is to assess the effect of PFG loss on soil microbial community composition and litter decomposition rate. To this end, we quantified responses of phospholipid fatty acid (PLFA) profiles of soil microbial communities and decomposition rates of leaf litter from grasses, forbs, and grass–forb mixtures to PFG loss by removing grasses, or forbs, or both grasses and forbs in a steppe vegetation in Inner Mongolia. We hypothesized that (1) PFG loss would have rapid effects on soil microbial biomass by reducing soil microbial biomass carbon (SMBC) and nitrogen (SMBN), the amount of bacterial and fungal PLFAs and on soil microbial community composition by altering the ratio of fungal to bacterial PLFAs; and (2) PFG loss would reduce decomposition rates of leaf litter from grasses, forbs, and grass–forb mixtures.

## Materials and Methods

### Study Site

This study was conducted at the Duolun Restoration Ecology Experimentation and Demonstration Station of the Institute of Botany, the Chinese Academy of Sciences, located in the southeast of Inner Mongolia, northern China (42°02’N, 116°16’E). Elevation is about 1,350 m above sea level. Long-term mean annual temperature at this site is 2.1°C, with monthly mean temperature ranging from -17.5°C in January to 18.9°C in July. Mean annual precipitation is about 380 mm, with 90% of the precipitation falling in the growing season between May and October.

Soil type is classified as chestnut soil (Chinese Classification) or Calcic Luvisols according to the FAO Classification (FAO, 1988). Surface soils (0–10 cm) are composed of 63% sand, 20% silt, and 17% clay. Soil pH is about 7.2, and soil bulk density is about 1.3 g cm^-3^. The native vegetation is represented by typical steppe communities. Dominant grasses at this site include *Leymus chinensis, Stipa capillata*, and *Agropyron cristatum*, and dominant forbs include *Artemisia frigida, Artemisiadalai lamae, Cymbaria dahurica, Potentilla bifurca*. Total vegetation cover ranges from 85 to 90%. The relative proportion of grass cover to total vegetation cover varies annually, but ranges from 30 to 60% (Song et al., 2011).

### Experimental Design

In early May of 2013, 20 plots (2 m × 3 m) were established at this study site. These plots were evenly spaced, separated by 1-m buffer strips, and arranged in four rows (five plots per row). All species in these plots were categorized as grasses and forbs based on their morphology. Treatments involved the removal of all grasses (hereafter: forbs only), all forbs (hereafter: grasses only), both forbs and grasses (hereafter: bare ground), and no removal (hereafter: control). Aboveground parts of plants were removed by clipping. To minimize the physical disturbance to soils, roots attached only to the base (or crown) of grasses and/or forbs were removed carefully. All treatments were arranged as a completely randomized design with five replications. Targeted plants established during the course of this study were removed periodically.

### Field Sampling and Lab Analyses

Soil temperature for each plot was recorded by temperature sensors with data loggers during the growing season of 2013 from mid-May to mid-October. All data-loggers were buried to a depth of 0–5 cm. Light intensity was measured for each plot in mid-May, mid-July, and mid-October (ca. 12:00–1:00 pm). In mid-July and mid-October, above- and below-ground plant biomass were estimated. Aboveground plant biomass was estimated by clipping all living parts of plants using a 1-m × 0.5-m quadrat within each plot. At each plot, five randomly located soil cores (10 cm in length and 8 cm in diameter) were collected for estimating belowground plant biomass. Roots were separated from soils by washing. All samples were oven-dried at 65°C to constant mass.

Soil samples for soil microbial community composition and other soil physiochemical properties were collected in mid-May, mid-July, and mid-October. At each plot, three randomly located soil cores (10 cm in length × 3 cm in diameter) were collected and composited. Soil samples were passed through a 2 mm sieve to remove coarse organic matters and transported with a portable ice box and stored at 4°C before analysis. Additional three soil cores within each plot were sampled and composited for estimating soil-water content by oven-drying samples at 105°C for 24 h.

A subsample of fresh soil was used to determine SMBC and SMBN using the fumigation-extraction method (Vance et al., 1987). Briefly, 20-g (dry weight equivalent) soil samples were fumigated with CH_3_Cl for 24 h and extracted with 0.5 M K_2_SO_4_. Equivalent samples of untreated soils were also extracted with 0.5 K_2_SO_4_. Extractions were filtered through 0.45-μm filters and analyzed for C and N concentrations with a Multi 3100 N/C TOC analyzer (Analytik Jena, Germany). SMBC and SMBN were calculated as the differences in extractable C and N concentrations between the fumigated and untreated soil samples using conversion factors (*k*_c_ and *k*_n_) of 0.38 and 0.45 (Lovell et al., 1995), respectively.

The microbial community structure in soil samples was assessed by the analysis of PLFAs (Bossio and Scow, 1998). Briefly, approximately 8-g (dry weight equivalent) soil samples were extracted twice using a single-phase chloroform/methanol/citrate buffer system (1:2:0.8 v/v/v; pH 4.0). The phospholipids were then separated from glycolipids and neutral lipids using silica acid columns and methylated using mild alkaline methanolysis. After methylation of phospholipids, PLFAs were then separated using a Gas Chromatograph (Agilent 6850, United States) and identified according to the standard protocol of the Sherlock Microbial Identification System V4.5 (MIDI). The abundance of individual fatty acids was determined as relative ng per g of dry soil and standard nomenclature was used. The fatty acids14:00, i14:00, 15:00, i15:0, a15:0, 16:00, i16:0, 16:1ω7c, i17:0, a17:0, 17:1ω8c, 18:1ω5c, 18:1ω9, 17:0cy, and 19:0cy were chosen to represent the PLFAs of the bacterial group, and fungi were considered to be represented by the PLFAs 16:1ω5c, 18:2ω6, 9c, and 18:1ω9c (Frostegård and Bååth, 1996; Bossio and Scow, 1998). All of the PLFAs mentioned above were used to calculate the total PLFAs of each soil microbial community. The ratio of fungal to bacterial PLFAs was also included in the analysis. This ratio has often been used as an indicator for changes in the soil microbial community composition (Bardgett et al., 1999).

Subsamples of sieved soils (20 g) collected in mid-July were air-dried and then pulverized. Soil organic carbon (SOC) concentration was determined by oxidation reduction titration of FeSO_4_ after distasting 0.2-g soil samples by K_2_CrO_7_–H_2_SO_4_ (Nelson and Sommers, 1996). To determine total soil nitrogen (TN), 1-g soil samples were digested using the Kjeldahl acid-digestion method (Bremner, 1996), and further analyzed on an auto-analyzer (Kjeltec 2200 Auto Distillation Unit, FOSS, Sweden). Soil total phosphorus (STP) concentration was determined using the molybdenum blue colorimetric method (Murphy and Riley, 1962), with a UV/visible spectrophotometer after digesting 1-g soil samples with H_2_SO_4_–H_2_O_2_ (Shimadzu UV-2550, Kyoto, Japan).

Litter decomposition rates were determined using the litter-bag method (Berg et al., 1993; Zhou et al., 2015). Briefly, senescent aboveground litter was collected from an adjacent field, oven-dried (65°C), and divided into litter from forbs and grasses. A 3-g sample of oven-dried litter (forbs only, grasses only, or grass–forb mixtures in a ratio of 1:1) was placed in each of 300 10 cm × 15 cm nylon bags (1-mm mesh size). At each plot, litterbags from all three litter types (i.e., forbs only, grasses only, and grass–forb mixtures) were randomly placed on the surface of the soil in mid-May of 2013 and retrieved monthly (i.e., mid-June, mid-July, mid-August, mid-September, and mid-October). In total, 300 litterbags were placed (3 litter types × 5 months × 20 plots). In the laboratory, retrieved litter was carefully washed to remove adhering soil particles and oven-dried (65°C) for biomass. We used the following exponential function: *Y_t_* = *Y*_0_ × e^-kt^ (Olson, 1963) to determine the decay constant (k). Where *Y*_0_ is the initial quantity of litter material, and *Y_t_* is the amount left at time *t*. Initial carbon, nitrogen, and phosphorous concentrations of each litter category were measured using the same methods for soils.

### Data Analyses

Two-way ANOVA was used to test for the effects of PFG loss, sampling time, and their interaction on measured soil variables, and used to test for the effects of PFG loss, litter type (i.e., grasses, forbs, grass–forb mixtures), and their interaction on litter decay constant and mass loss. Differences in means of variables among different treatments were compared by one-way ANOVA following with Tukey’s HSD comparison. A cutoff of *p* < 0.05 was used to indicate significance. All statistical analyses were performed on SPSS 17.0 (SPSS, Chicago, IL, United States).

## Results

**FIGURE 1:**
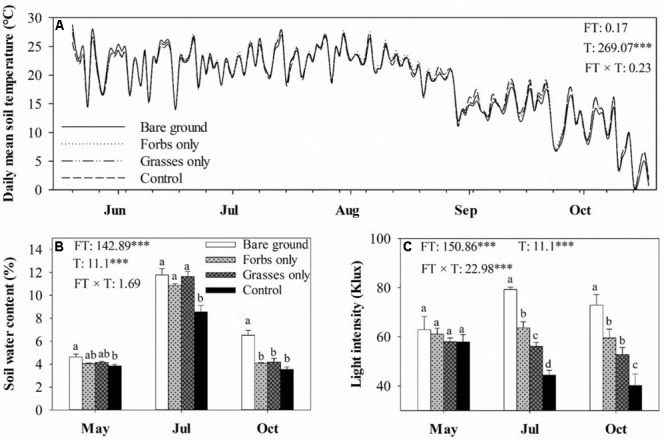
Soil daily mean temperature **(A)**, soil monthly water content **(B)**, and light intensity in May, July, and October **(C)** for each plant functional group loss treatment. Values are means ± SE, *n* = 5. For soil daily mean temperature, only means are presented. Significant differences (*p* < 0.05) between means for each treatment are indicated with different letters. Inserts are results from two-way ANOVA of plant functional group loss (FT), sampling time (T), and their interaction (FT × T). ^∗∗∗^ represents significant differences at *p* < 0.001.

**FIGURE 2:**
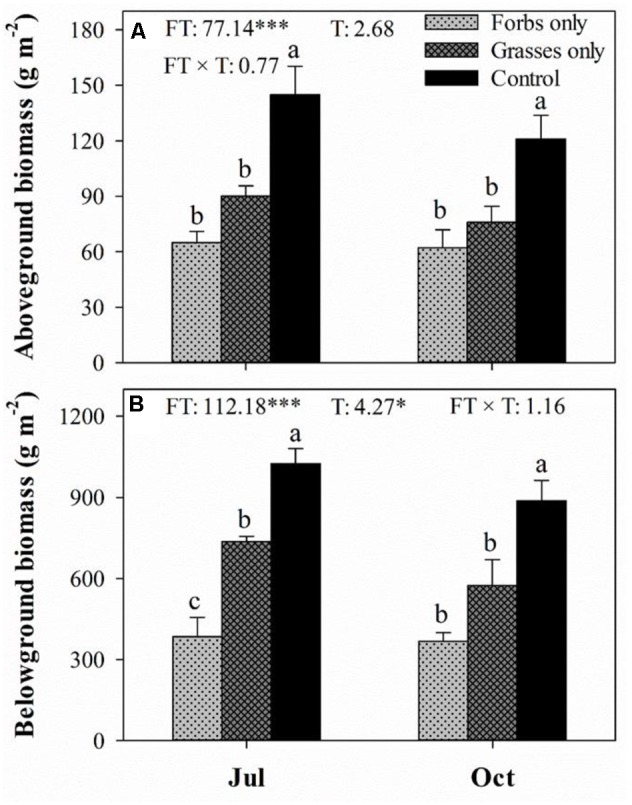
Aboveground **(A)** and belowground **(B)** biomass (g m^-2^) for each plant functional group loss treatment in July and October 2013. Values are means ± SE, *n* = 5. Significant differences (*p* < 0.05) between means for each treatment are indicated with different letters. Inserts are results from two-way ANOVA of plant functional group loss (FT), sampling time (T), and their interaction (FT × T). ^∗^ and ^∗∗∗^ represent significant differences at *p* < 0.05 and *p* < 0.001.

Plant functional group loss did not affect daily mean soil temperature (**Figure 1A**). Averaged soil temperatures for bare ground, forbs only, grasses only, and control plots from mid-May to mid-October were 18.8, 19.1, 18.8, 18.8°C, respectively. Soil water content peaked in mid-July, and differed significantly among treatments (**Figure 1B**, *p* < 0.001). PFG loss increased soil water content, especially for plots with the removal of both forbs and grasses (**Figure 1B**). Light intensity differed significantly among treatments and sampling time (**Figure 1C**, *p* < 0.001). PFG loss significantly increased light intensity in mid-July and mid-October (**Figure 1C**). PFG loss did not affect SOC, STP, soil C:N and C:P ratios (**Table 1**). Soils from bare ground plots had significant lower STN than from other plots (**Table 1**). Belowground biomass was almost an order of magnitude higher than aboveground biomass in this steppe vegetation, and PFG loss significantly reduced both above- and below-ground biomass (**Figure 2**, *p* < 0.001). Plots with only forbs had lower both above- and below-ground biomass than plots with only grasses (**Figure 2**).

**Table 1:** Carbon (C), nitrogen (N), and phosphorus (P) concentrations, C:N and C:P ratios for soil from each plant functional group loss treatment and for litter from forbs, grasses, and grass–forb mixtures.

	C (g kg^-1^)	N (g kg^-1^)	P (g kg^-1^)	C:N ratio	C:P ratio
Soil				
Bare ground	12.0 (0.2)^a^	1.29 (0.02)^b^	0.30 (0.01)^a^	9.3 (0.2)^a^	39.7 (2.2)^a^
Forbs only	13.0 (1.2)^a^	1.45 (0.07)^ab^	0.33 (0.01)^a^	9.1 (0.9)^a^	39.5 (4.0)^a^
Grasses only	13.3 (1.1)^a^	1.50 (0.08)^ab^	0.34 (0.02)^a^	9.0 (0.9)^a^	39.4 (3.7)^a^
Control	14.1 (1.1)^a^	1.62 (0.03)^a^	0.36 (0.01)^a^	8.7 (0.7)^a^	39.0 (2.4)^a^
Litter				
Forbs	364.0 (15.1)^a^	9.19 (0.32)^a^	0.65 (0.03)^a^	39.8 (2.8)^a^	561.5 (44.6)^a^
Grasses	411.0 (24.4)^a^	8.85 (0.74)^a^	0.55 (0.03)^a^	46.8 (2.9)^a^	758.6 (71.1)^a^
Mixtures	375.8 (5.6)^a^	9.03 (0.37)^a^	0.62 (0.02)^a^	42.8 (1.6)^a^	608.5 (22.5)^a^

*Values are means ± SE, *n* = 5. Significant differences (*p* < 0.05) between means for each treatment are indicated with different letters according to Tukey’s HSD comparison.*

**FIGURE 3:**
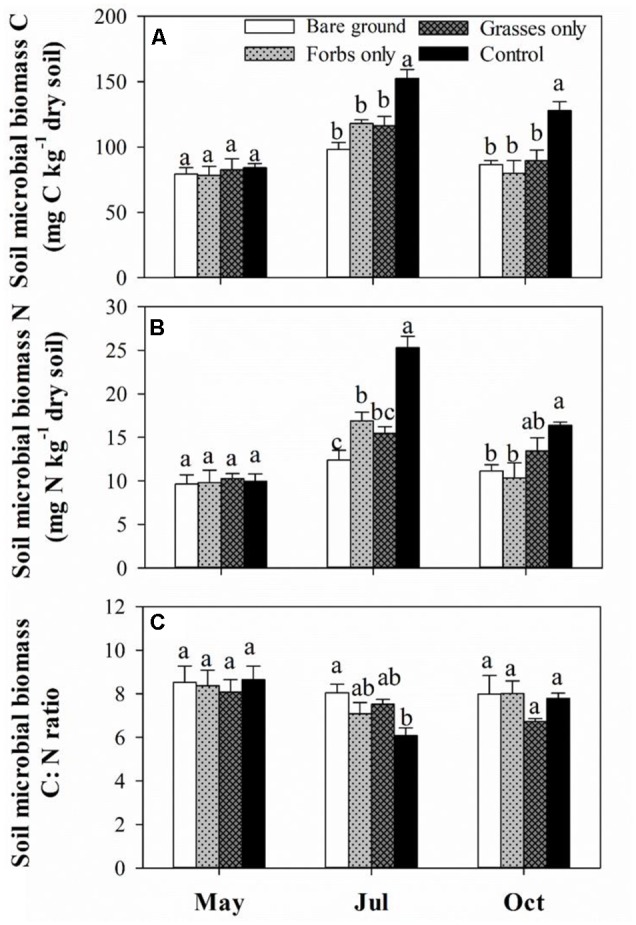
Soil microbial biomass C **(A)**, N **(B)**, and C:N ratio **(C)** for each plant functional group loss treatment in May, July, and October 2013. Values are means ± SE, *n* = 5. Significant differences (*p* < 0.05) between means for each treatment are indicated with different letters.

**FIGURE 4:**
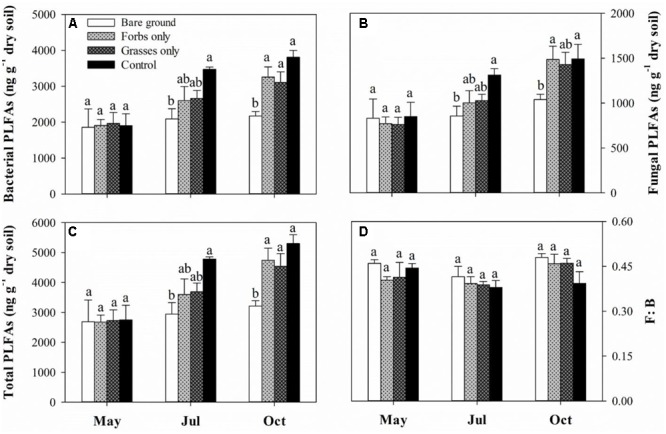
Soil bacterial PLFAs **(A)**, fungal PLFAs **(B)**, total PLFAs **(C)**, and F:B ratio **(D)** for each plant functional group loss treatment in May, July, and October 2013. F:B ratio indicates the ratio of fungal to bacterial PLFAs. Values are means ± SE, *n* = 5. Significant differences (*p* < 0.05) between means for each treatment are indicated with different letters.

Soil microbial biomass carbon and SMBN peaked in mid-July and differed significantly among treatments (**Figures 3A,B** and **Table 2**, *p* < 0.001). Immediately after the removal of PFGs (i.e., mid-May), there was no significant difference in SMBC and SMBN among treatments (**Figures 3A,B**). However, PFG loss significantly reduced SMBC and SMBN in mid-July and mid-October (**Figures 3A,B**). Both treatment and sampling time had no effect on the ratio of SMBC:SMBN (**Table 2**), although the ratio of SMBC:SMBN was significantly lower in control plots than other plots in mid-July (**Figure 3C**). The removal of PFGs had significant effects on the amount of bacterial, fungi, and total PLFAs (**Table 2**, *p* < 0.05). Although there was no significant difference at the initial period of this study (i.e., mid-May), PFG loss reduced the amount of bacterial, fungi, and total PLFAs in mid-July and mid-October (**Figures 4A–C**). Sampling time had significant effects on the amount of bacterial, fungi, and total PLFAs (**Table 2**, *p* < 0.001) which increased with time during the growing season of 2013 (**Figures 4A–C**). PFG loss had no effects on the ratio of fungal:bacterial PLFAs (**Table 2** and **Figure 4D**), whereas the ratio of fungal:bacterial PLFAs was lowest in mid-July (**Table 2** and **Figure 4D**).

**Table 2:** Results (*F*-values) of two-way ANOVA on the effects of plant functional group loss (FT), sampling time (T), and their interaction on soil microbial biomass carbon (SMBC), soil microbial biomass nitrogen (SMBN), SMBC:SMBN ratio, the amount of bacterial, fungal, and total PLFAs, and the ratio of bacterial to fungal PLFAs (F:B ratio).

Factors	FT	T	FT × T
SMBC	14.66^∗∗∗^	33.28^∗∗∗^	3.04^∗^
SMBN	11.05^∗∗∗^	30.50^∗∗∗^	4.25^∗∗^
SMBC:SMBN ratio	0.47	1.98	1.04
Bacterial PLFAs	5.56^∗∗^	19.08^∗∗∗^	1.49
Fungal PLFAs	6.31^∗∗^	17.40^∗∗∗^	1.61
Total PLFAs	2.98^∗^	219.35^∗∗∗^	1.15
F:B ratio	1.65	4.34^∗^	0.88

*^∗^, ^∗∗^, and ^∗∗∗^ represent significant differences at *p* < 0.05, *p* < 0.01, and *p* < 0.001.*

**FIGURE 5:**
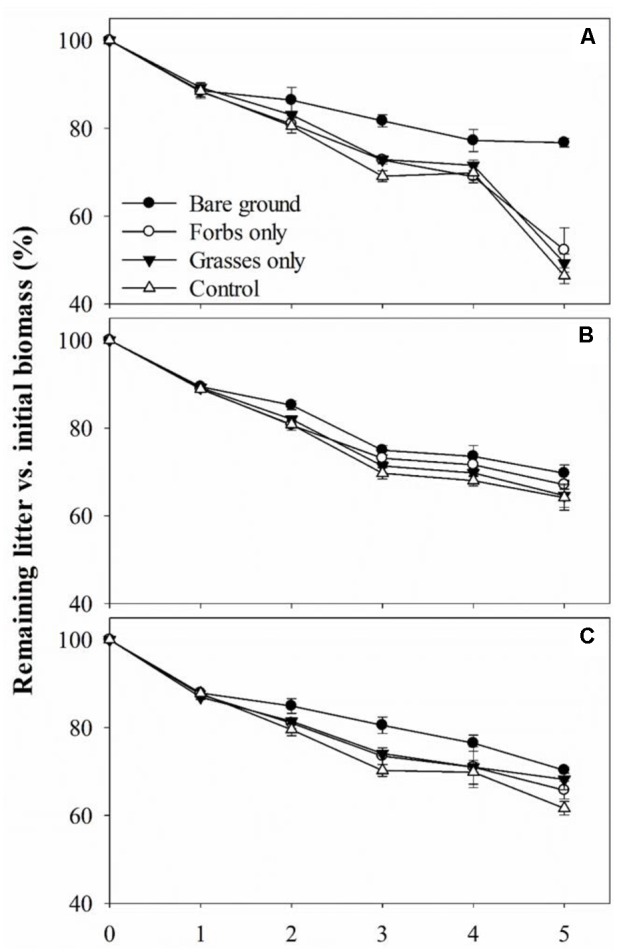
Remaining forbs litter **(A)**, grasses litter **(B)**, and grass–forb mixtures litter **(C)** versus initial mass for each plant functional group loss treatment from May to October 2013. Values are means ± SE, *n* = 5.

No differences were found in litter C, N, and P concentrations, C:N and C:P ratio for grasses, forbs, and grass–forb mixtures (**Table 1**). Results from the litter-bag method indicated that litter monthly mass loss ranged from 7.0 to 10.0%, and decay constant ranged from 0.08 to 0.12 (**Table 3**). PFG loss had significant effects on litter monthly mass loss and decay constant (**Table 4**, *p* < 0.01). The removal of PFGs decreased litter monthly mass loss and decay constant during the growing season of 2013, regardless the type of litter (**Figure 5** and **Table 3**). However, such decrease was significant only under the extreme scenario when both forbs and grasses were removed (**Figure 5** and **Table 3**). Litter type had no significant effects on monthly mass loss and decay constant (**Table 4**).

**Table 3:** Mean monthly decay constant (*k*) and mass loss (%) of forb, grass, and grass–forb mixture litter in each plant functional group loss treatment from May to October 2013.

	*k*	*R*^2^	*p*	Mass loss (% month^-1^)
**Forb litter**			
Bare ground	0.09 (0.00)^b^	0.81	<0.0001	7.80 (0.37)^b^
Forbs only	0.10 (0.00)^a^	0.88	<0.0001	8.79 (0.31)^ab^
Grasses only	0.10 (0.00)^a^	0.85	<0.0001	8.66 (0.39)^ab^
Control	0.11 (0.00)^a^	0.85	<0.0001	9.20 (0.34)^a^
**Grass litter**			
Bare ground	0.08 (0.01)^b^	0.55	<0.0001	6.93 (0.65)^b^
Forbs only	0.11 (0.01)^a^	0.84	<0.0001	9.09 (0.30)^ab^
Grasses only	0.11 (0.01)^a^	0.79	<0.0001	9.50 (0.43)^a^
Control	0.12 (0.01)^a^	0.84	<0.0001	9.96 (0.32)^a^
**Grass–forb mixture litter**			
Bare ground	0.08 (0.01)^b^	0.80	<0.0001	7.59 (0.53)^b^
Forbs only	0.10 (0.01)^a^	0.86	<0.0001	8.91 (0.50)^ab^
Grasses only	0.10 (0.01)^a^	0.69	<0.0001	8.98 (0.51)^ab^
Control	0.11 (0.00)^a^	0.84	<0.0001	9.51 (0.42)^a^

*Values are means ± SE, *n* = 5. Significant differences (*p* < 0.05) between means for each functional type loss treatment are indicated with different letters according to Tukey’s HSD comparison.*

**Table 4:** Results (*F*-values) of two-way ANOVA on the effects of plant functional group loss (FT), litter type (LT), and their interaction on decay constant (*k*) and mass loss (%).

Factors	FT	LT	FT × LT
*k*	19.97^∗∗^	1.52	1.56
Mass loss	13.15^∗∗^	0.35	0.86

*^∗∗^ represents significant differences at *p* < 0.01.*

## Discussion

Our study provides robust evidence that PFG loss in this Inner Mongolian steppe vegetation has rapid effects on soil microbial biomass as SMBC, SMBN, and the amount of bacterial, fungal, and total PLFAs were significantly reduced after the removal of PFGs (**Figures 3, 4** and **Table 2**). However, we found that soil microbial community composition was not affected by PFG loss as the ratio of fungal to bacterial PLFAs was not altered by the removal of PFGs throughout the growing season of 2013 (**Figure 4** and **Table 2**). These results lead us to reject our first hypothesis that PFG loss would alter soil microbial composition. However, it should be noticed that results from this study were based on a coarse measure of soil microbial composition (i.e., PLFAs); a finer measure (e.g., 16s rRNA) of soil microbial taxa or functional groups would further enhance our understanding and assessment of responses of soil microbial composition to PFG loss in grasslands.

Despite soil microbial biomass remaining unaffected in May 2013 shortly after the removal of PFGs, a negative effect of PFG loss on soil microbial biomass was observed in July and continued to the end of growing season (**Figures 3, 4**). Reduced soil microbial biomass response to PFG loss in this study is consistent with other short-term and long-term studies (Spehn et al., 2000; Zak et al., 2003; Loranger-Merciris et al., 2006; Eisenhauer et al., 2010; Scherber et al., 2010; Chen et al., 2016). In this study, PFG loss reduced soil water content and increased solar radiation exposures (**Figure 1**) which, to some extent, may indirectly affect soil microbial activity and abundance. However, soil microorganisms are mostly heterotrophs that depend on plant-derived organic resources, such as leaf and root litter and root exudates (Zak et al., 2003; Wardle et al., 2004; Bardgett and Wardle, 2010). Thus, we suggest that observed decreases in soil microbial biomass in response to PFG loss in this steppe vegetation likely result from declines in quantity and/or quality of plant-derived organic materials. For example, Spehn et al. (2000) showed that microbial biomass decreased with decreasing plant species in a Swiss grassland ecosystem and associated this decline in soil microbial biomass to the decrease of plant biomass production. In the present study, the removal of PFG significantly reduced both above- and below-ground plant biomass (**Figure 2**) and is likely to reduce the quantity of organic matter entering the soils through litterfall and root turnover. In addition, species/PFG loss may lead to lower biochemical diversity of plant-derived organic resources, therefore supporting a lower degree of soil microbial activity and reducing soil microbial biomass (Loranger-Merciris et al., 2006; Milcu et al., 2006; Eisenhauer et al., 2010).

In contrast to reduced soil microbial biomass in response to PFG loss, there was no effect of PFG loss on soil microbial community composition with respect to the relative proportion of fungi to bacteria (**Figure 4**). This might be ascribed to the short duration of this study. Several short-term studies also found that plant diversity or PFG richness did not affect soil microbial community composition (Gastine et al., 2003; Marshall et al., 2011). In a long-term study (i.e., 6 years) in the framework of the Jena Experiment, Eisenhauer et al. (2010) suggests that there may be substantial time lags in the establishment of plant species richness effects on soil microbial communities. This time lag can be as long as 4 years after the manipulation of plant species richness and PFG richness (Eisenhauer et al., 2010). Although soil microbial community composition (as indicated by the ratio of fungal to bacterial PFLAs) was insensitive to PFG loss in this short-term study, another 4-year PFG-removal experiment in the Inner Mongolian grassland found that the removal of perennial bunchgrasses or perennial rhizomes decreased the ratio of fungal to bacterial PLFAs by 9–12% (Chen et al., 2016). Chen et al. (2016) suggested that the shift from fungal-based to bacterial-based soil microbial community composition resulting from PFG loss could amplify nutrient losses and CO_2_ release.

Plant functional group loss reduced litter decomposition rates regardless the type of leaf litter (**Figure 5** and **Tables 3, 4**), thereby supporting our second hypothesis. However, litter type had no effects on litter decomposition rates (**Figure 5** and **Tables 3, 4**). As leaf litter traits differ among plant species and PFGs (Cornwell et al., 2008; Allison, 2012; Makkonen et al., 2012), the mainstream view is that species interactions in mixed-species litter are fairly common and cause distinct decomposition trajectories that differ from those expected from single-species litter (Hättenschwiler et al., 2005). For example, Gartner and Cardon (2004) have shown that mass loss is often increased when litters of different species are mixed and non-additive patterns of mass loss were observed in 67% of tested mixtures. In this present study, decomposition rates of leaf litter from grasses, forbs, and grass–forb mixtures were comparable and no synergistic or antagonistic interactions were found for grass–forb mixtures. This is probably due to the fact that there were no significant differences in chemical composition of leaf litter from grasses, forbs, and grass–forb mixtures (**Table 1**). For example, the C:N ratio of leaf litter, which is frequently used as an predictor for litter decomposition rate (Melillo et al., 1982; Enríquez et al., 1993; Aerts, 1997), was comparable among grasses, forbs, and grass–forb mixtures (**Table 1**).

As the present study excluded the potential effect of litter quality (as noted above) on litter decomposition rate, environmental conditions and decomposers are other two primary controls on it (Melillo et al., 1982; Aerts, 1997). Although soil temperatures were comparable among different treatments (**Figure 1**), temperatures at ground surface where litterbags were placed could have been higher in the bare ground treatment in comparison to the other treatments covered by vegetation. Higher ground surface temperatures and increased solar radiation exposures (**Figure 1**) could theoretically accelerate litter decomposition (King et al., 2012; Zhou et al., 2015). However, in this study, we observed that litter decomposition rates in the bare ground treatment, regardless of litter type, were lower than other treatments, suggesting that decreases in decomposition rate after PFG loss may be related to declines in the activity of decomposers after the removal of PFG. Soil fauna have been shown to affect the initial period of litter decomposition directly by selectively degrading plant detritus and indirectly by controlling soil microbial activity (Hättenschwiler and Gasser, 2005; Wardle et al., 2006). The activity of soil fauna is tightly linked to species/PFG richness as prior reports have demonstrated that species/PFG loss reduced the abundance and diversity of soil fauna (Gastine et al., 2003; Vos et al., 2011; Chen et al., 2016). In addition to soil fauna, microbially mediated litter decomposition may also contribute to the explanation of decreased litter decomposition rate after PFG loss (Butenschoen et al., 2011). Although soil microbial community composition remained unaffected, PFG loss had a rapid and negative effect on soil microbial activity in this steppe vegetation (**Figures 3, 4**). Significantly reduced soil microbial activity after PFG loss may influence the colonization of microorganisms and subsequent utilization of leaf litter, thereby retarding litter decomposition rate. These findings point to the necessity of developing a framework connecting plant diversity, soil biota, and litter decomposition to allow predicting the consequences of biodiversity loss for soil nutrient cycling.

In summary, we found that PFG loss in this steppe vegetation had a rapid and negative effect on soil microbial activity by reducing SMBC, SMBN, and the amounts of fungal, bacterial, and total PLFAs. However, soil microbial community composition as indicated by the ratio of fungal to bacterial PLFAs was insensitive to PFG loss due to the short duration of this study. PFG loss reduced decomposition rates of leaf litter from grasses, forbs, and grass–forb mixtures. Our findings indicate that PFG loss and the associated declines in plant biomass can lead to rapid effects on soil microbial activity, thereby affecting litter decomposition and soil nutrient cycling. Our results also highlight that the assessment of plant–microbe interactions in soils is an integral component of ecosystem functioning response to biodiversity loss.

## Author Contributions

CX formulated the original idea and developed methodology. JS performed sample processing. JS and FY performed statistical analysis. YZ wrote the first draft of the manuscript. All authors contributed critically to revision.

## Conflict of Interest Statement

The authors declare that the research was conducted in the absence of any commercial or financial relationships that could be construed as a potential conflict of interest.
